# Supplementary material for: Content-rich biological network constructed by mining PubMed abstracts
Source: BMC Bioinformatics. 2004 Oct 8;5:147. doi: 10.1186/1471-2105-5-147 (PMC528731; doi:10.1186/1471-2105-5-147)
Supplement: Additional File 2 — The original results of the above study (non-essential files are deleted to keep the file size under the limit set by BMC bioinformatics). [file 1471-2105-5-147-S2.bz2 › chilibotAdditionalFile2/dip20/506ID11278309E10794/html/AR_CAV1.html]

 


 **AR** and **CAV1** 
  
Found 18 abstracts in PubMed, retrieved 18.  
 

 What does Google say? 
 PDF only 
| .edu only 

---

**Interactive relationship** (e.g. stimulation, inhibition, etc)

**Inhibitory relationship**- CONCLUSIONS AECA may be important in the mediation of humoral  **AR** , may decrease allograft survival, and may identify a high risk group for CAV  [ **CAV1** ] .  Ref: 10030283 Transplantation, 1999
- The sequestration of alpha2A  **AR**  was partly inhibited after sucrose pretreatment but could be further reduced after inhibiting the formation of both clathrin coated and caveolin  [ **CAV1** ]  vesicles by combined pretreatment with hyperosmotic sucrose and filipin.  Ref: 12800978 Eur J Cell Biol, 2003
**Neutral relationship**- Co immunoprecipitation and glutathione S transferase fusion protein pull down experiments demonstrate that interaction between  **AR**  and caveolin 1  [ **CAV1** ]  is an androgen dependent process, offering further evidence for a physiological role of this interaction.  Ref: 11278309 J Biol Chem, 2001
- Caveolin 1  [ **CAV1** ]  interacts with androgen receptor  [ **AR** ] .  Ref: 11278309 J Biol Chem, 2001
- Immunoblot analysis of membrane fractions indicated that beta  **AR**  and AC6 are expressed in fractions enriched in caveolin  [ **CAV1** ]  3 and morphologic caveolae.  Ref: 10779394 Mol Pharmacol, 2000
- Conversely, down regulation of caveolin 1  [ **CAV1** ]  expression by a caveolin 1  [ **CAV1** ]  antisense expression construct can down regulate ligand dependent  **AR**  activation.  Ref: 11278309 J Biol Chem, 2001
- Antisense and pharmacological experiments provided additional evidence that the sequential activation of PKCepsilon, mitogen activated protein kinases, c Myc, and androgen receptor  [ **AR** ]  signaling drove the downstream expression of caveolin 1  [ **CAV1** ]  in CWR R1 cells.  Ref: 12185081 J Biol Chem, 2002
- Evidence for beta 2  **AR**  localization to caveolae includes co localization by confocal imaging, co immunoprecipitation of the beta 2  **AR**  and caveolin  [ **CAV1** ]  3, and co migration of the beta 2  **AR**  with a caveolin  [ **CAV1** ]  3 enriched membrane fraction.  Ref: 12097322 J Biol Chem, 2002
- Using a mammalian two hybrid assay system, we determine that the NH 2 terminus region of caveolin 1  [ **CAV1** ]  is responsible for the interaction with both the NH 2 terminal domain and the ligand binding domain of  **AR** .  Ref: 11278309 J Biol Chem, 2001

**Non-interactive relationship** (e.g. studied together, co-existance, homology, etc.)

- Based on a previous study in the American and Brazilian population, it has been suggested that CAV  [ **CAV1** ]  3 mutations might also cause  **AR**  LGMD.  Ref: 11251997 Am J Med Genet, 2001
- Association between these two molecules is also demonstrated by co localization of  **AR**  with caveolin  [ **CAV1** ]  rich, low density membrane fractions isolated by an equilibrium sucrose gradient centrifugation method.  Ref: 11278309 J Biol Chem, 2001
- Expression of caveolin  [ **CAV1** ] , androgen receptor  [ **AR** ]   **AR**  and prostate specific antigen PSA was also examined after reintroduction of androgen.  Ref: 10420156 Prostate, 1999
- Thus, the players high playing arm BMC was due to increases in the Tot. **Ar** , M.Cav  [ **CAV1** ] . **Ar** , Co. **Ar** , and CW.Th.  Ref: 10962345 Bone, 2000
- The beta 2  **AR**  stimulated increase in the myocyte contraction rate is increased by approximately 2 fold and markedly prolonged by filipin, an agent that disrupts lipid rafts such as caveolae and significantly reduces co immunoprecipitation of beta 2  **AR**  and caveolin  [ **CAV1** ]  3 and co migration of beta 2  **AR**  and caveolin  [ **CAV1** ]  3 enriched membranes.  Ref: 12097322 J Biol Chem, 2002
- The aim was to determine the incidence of AECA formation after transplantation and their association with different types of  **AR** , graft survival, and development of cardiac allograft vasculopathy CAV  [ **CAV1** ] .  Ref: 10030283 Transplantation, 1999
- The side to side M.Cav  [ **CAV1** ] . **Ar**  difference was significant at the proximal humerus 19% and radial shaft 29%.  Ref: 10962345 Bone, 2000
- In this study, we investigate the potential interactions between  **AR**  signaling and caveolin 1  [ **CAV1** ]  and demonstrate that overexpression of caveolin 1  [ **CAV1** ]  potentiates ligand dependent  **AR**  activation.  Ref: 11278309 J Biol Chem, 2001
- Using radioligand receptor binding assay and immunoblot analysis, we examined the distribution and the amount of alpha1  **AR**  and caveolin  [ **CAV1** ] .  Ref: 11530104 Cardiovasc Res, 2001
- The pQCT variables analyzed were bone mineral content BMC, total cross sectional area of bone Tot. **Ar** , cross sectional area of the marrow cavity M.Cav  [ **CAV1** ] . **Ar** , cortical bone Co. **Ar**  and trabecular bone Tr. **Ar** , volumetric density of cortical Co.Dn and trabecular Tr.  Ref: 10962345 Bone, 2000
- Caveolin 1  [ **CAV1** ] , androgen receptor  [ **AR** ] , c Myc, and protein kinase Cepsilon PKCepsilon proteins are overrepresented in most advanced prostate cancer tumors.  Ref: 12185081 J Biol Chem, 2002
